# Quorum sensing regulates a polysaccharide biosynthesis gene to control cell aggregation in *Paracoccus denitrificans*

**DOI:** 10.1093/ismeco/ycaf198

**Published:** 2025-10-31

**Authors:** Kana Morinaga, Kohei Takahashi, Ryo Nagasawa, Wenzhi Tao, Shinya Sugimoto, Nozomu Obana, Nobuhiko Nomura, Andrew S Utada, Masanori Toyofuku

**Affiliations:** Graduate School of Life and Environmental Sciences, University of Tsukuba, Tennodai, Tsukuba, Ibaraki 305–8572, Japan; Graduate School of Life and Environmental Sciences, University of Tsukuba, Tennodai, Tsukuba, Ibaraki 305–8572, Japan; Graduate School of Life and Environmental Sciences, University of Tsukuba, Tennodai, Tsukuba, Ibaraki 305–8572, Japan; Graduate School of Life and Environmental Sciences, University of Tsukuba, Tennodai, Tsukuba, Ibaraki 305–8572, Japan; Department of Bacteriology and Jikei Center for Biofilm Research and Technology, The Jikei University School of Medicine, Nishi-Shimbashi, Minato-ku, Tokyo 105-8461, Japan; Transborder Medical Research Center, Faculty of Medicine, University of Tsukuba, Tennodai, Tsukuba, Ibaraki 305–8577, Japan; Microbiology Research Center for Sustainability (MiCS), University of Tsukuba, Tennodai, Tsukuba, Ibaraki 305–8572, Japan; Microbiology Research Center for Sustainability (MiCS), University of Tsukuba, Tennodai, Tsukuba, Ibaraki 305–8572, Japan; Institute of Life and Environmental Sciences, University of Tsukuba, Tennodai, Tsukuba, Ibaraki 305–8572, Japan; Tsukuba Institute for Advanced Research, University of Tsukuba, Tennodai, Tsukuba, Ibaraki 305-8577, Japan; Microbiology Research Center for Sustainability (MiCS), University of Tsukuba, Tennodai, Tsukuba, Ibaraki 305–8572, Japan; Institute of Life and Environmental Sciences, University of Tsukuba, Tennodai, Tsukuba, Ibaraki 305–8572, Japan; Tsukuba Institute for Advanced Research, University of Tsukuba, Tennodai, Tsukuba, Ibaraki 305-8577, Japan; Microbiology Research Center for Sustainability (MiCS), University of Tsukuba, Tennodai, Tsukuba, Ibaraki 305–8572, Japan; Institute of Life and Environmental Sciences, University of Tsukuba, Tennodai, Tsukuba, Ibaraki 305–8572, Japan; Tsukuba Institute for Advanced Research, University of Tsukuba, Tennodai, Tsukuba, Ibaraki 305-8577, Japan

**Keywords:** quorum sensing, biofilm, cellular aggregation, cell-to-cell adhesion, cell-surface adhesion, non motile species, *Paracoccus denitrificans*

## Abstract

Most biofilm research has centered on a few motile model organisms that form thick biofilms. During dispersal, motile bacteria actively escape through motility; however, nonmotile species likely rely on the modulation of adhesive interactions to regulate detachment. In this study we show that the nonmotile soil organism *Paracoccus denitrificans* maintains thin biofilms through constitutive quorum sensing (QS). We show that a LuxI/LuxR-type QS system actively suppresses cell-to-cell adhesion to maintain a thin biofilm architecture, while maintaining a basal level of cell-surface adhesion. We identified a polysaccharide biosynthesis gene, *pxm*, which is essential for cellular aggregation. Disruption of QS leads to thick biofilm formation with necrotic cores, mediated by Pxm-dependent production. Using microfluidic chambers, we examined interactions between Pxm and BapA adhesion factors under varying spatial confinement. Our findings reveal that QS-dependent suppression of adhesion factors enables *P. denitrificans* to form thin, loosely connected biofilms that promote nutrient access and dispersal, providing an ecological advantage in spatially confined environments such as soil or aquatic sediments.

## Introduction

In nearly all environments, most bacteria form biofilms [1]. Much of our understanding of model biofilm development derives from studies using motile organisms such as *Pseudomonas aeruginosa* and *Vibrio cholerae* [2–5]. In contrast, much less is known about biofilm development using nonmotile bacteria. The nonmotile *P. denitrificans* is a facultative anaerobe that plays an important role as a denitrifier in the global nitrogen cycle. This Gram-negative bacterium is found primarily in environments that experience dynamic changes in oxygenation, such as in wet soils and in wastewater treatment plants. Although it has been used as a model organism to study its respiration pathways and ability for heterotrophic nitrification-aerobic denitrification [6–8], the mechanisms underlying its biofilm formation remain poorly understood [9, 10].

The canonical biofilms formed by *P. aeruginosa* and *V. cholerae* are three-dimensional structures composed of densely packed bacteria encased in a matrix of self-secreted extracellular polymeric substances (EPS), composed of polysaccharides, proteins, and extracellular DNA (eDNA). EPS functions as a molecular glue, adhering the community to a surface and to each other. Although the dense packing of cells within biofilms enables resource sharing and protection against external stresses such as dehydration and predation [11–13], it also leads to nutrient depletion, resource competition, and accumulation of metabolic waste [14–16]; these stressors can lead to cell death. The trade-offs between the benefits and drawbacks of forming dense communities likely dictate the observed biofilm morphology in specific environments for different bacterial species.

To mediate stress from community living, bacteria possess regulatory mechanisms that control biofilm dispersal. Quorum sensing (QS) is a form of intercellular communication using diffusible signals that provides information on the local cell density, enabling the community to synchronize behavior [17–19]. Under sufficiently high cell densities, concentrations of QS signal molecules can reach critical levels, creating a cascade that up-regulates EPS-digestion factors, ultimately enabling active dispersal from the biofilm [14–16, 20–22].

However, *P. denitrificans* exhibit a markedly different strategy. In contrast to the thick biofilms formed by *P. aeruginosa* and *V. cholerae, P. denitrificans* form biofilms of only a few micrometers [10]. The maintenance of this thin biofilm architecture involves complex regulatory networks [9, 23], with the QS system playing a crucial role [18, 24]. Like many Gram-negative bacteria, *P. denitrificans* utilizes a LuxI/LuxR QS homolog system [25]; here, the LuxI homolog, PdnI, synthesizes N-acyl-homoserine-lactone (AHL) signaling molecules while the LuxR homolog, PdnR, serves as the cognate receptor [26–28] (see Supplementary Information for gene naming). In *P. denitrificans*, disruption or dysregulation of the QS system by deletion of *pdnI* or by overexpression of *pdnR*, respectively, leads to the formation of a thick biofilm with large aggregates in liquid culture [10, 24, 26, 28]. The aggregation factors responsible for these thick biofilms and their regulatory mechanisms remain unknown. Clarifying the key matrix components responsible for biofilm formation and their connection to QS in *P. denitrificans* will further advance our fundamental understanding of this important soil bacterium, may help clarify biofilm formation strategy in nonmotile organisms, and inform biotechnological applications aimed at controlling biofilm formation.

In this paper, we show that constitutive quorum sensing in *P. denitrificans* suppresses cell-to-cell adhesion factors, leading to the formation of thin biofilms. We identify a previously uncharacterized polysaccharide-biosynthesis gene in *P. denitrificans* i.e. necessary for cellular aggregation, which we name the *Paracoccus* extracellular matrix (*pxm*) gene. We test the roles of Pxm and the known surface-adhesion protein BapA to investigate biofilm formation under different degrees of confinement using microfluidic chambers. When unconfined, we show that Pxm overproducing strains form thick biofilms that develop necrotic cores. Secondarily, we find that Pxm overexpression plays a complementary role in surface attachment, restoring surface adhesion of mutants lacking BapA. In semi-confined conditions, Pxm and BapA overexpression synergistically lead to a near complete suppression of cell escape from microcolonies. Under high confinement, where only cell monolayers can form, we observe a convergence of microcolony phenotypes in all Pxm overexpression strains and WT.

## Material and methods

### Bacterial strains, growth conditions, and transposon mutant construction

We cultured *P. denitrificans* Pd1222 and all derived strains at 30 or 37°C in Tryptic Soy Broth (TSB) medium under continuous shaking at 190 rpm [26, 29]. *Escherichia coli* DH5a and S17–1 strains were cultured at 37°C in Lysogeny Broth (LB) medium under continuous agitation (190 rpm). When necessary, we supplemented the antibiotics kanamycin or rifampicin at 50 μg ml^−1^ and 100 μg ml^−1^, respectively. To construct mutants, complemented strains, and fluorescent strains, we followed a procedure described in the Supplementary Information. To construct our transposon library, we used a Tn5-carrying plasmid following the procedure described in the Supplementary Information. All of the strains, plasmids, and primers used in our tests are summarized in Tables S1 and S2.

### Concanavalin A staining

We cultured cell samples for Concanavalin A (ConA) staining by depositing 5 μL of overnight liquid culture on solid LB agar, which we incubated at 30°C for another 72 h. We then collected the colony from the plate using a loop and suspended it in 100 μL of PBS containing a ConA-Alexa594 (Thermo Fisher Scientific) at a final concentration of 50 μg ml^−1^. After incubating this suspension overnight, we washed the cells twice in PBS, deposited 1 μL on coverglass and acquired fluorescence and brightfield images.

### Scanning electron microscopy

We conducted scanning electron microscopy (SEM) on our bacteria using an S-4200 (Hitachi, Japan). To prepare samples for SEM, cells from 48 h liquid cultures were harvested, fixed, and then sputter coated with palladium/platinum using an ion coater E-1030 (Hitachi, Japan).

To fix the cells, we centrifuged the cells, discarded the supernatant, and resuspended in a fixing solution containing 3% volume/volume (v/v) glutaraldehyde dissolved in 0.1 M phosphate buffer saline (PBS) for 30 min. The solution was then replaced, and we soaked the cells overnight. The following day, we dehydrated the samples with sequential washes in 50%, 70%, 90%, and 100% v/v ethanol in deionized (DI) water, respectively. The samples were then stored in 100% ethanol overnight. The following day, in preparation for sputter coating, we dried the samples in liquid CO_2_ with an HPC-2 critical point dryer (Hitachi, Japan).

### Real-time PCR

To measure the expression levels of *pxm, bapA*, and *rpoZ*, we conducted real-time PCR (see Table S2). We began by collecting RNA from 16 h culture (OD_600_ = ~2) using the RNeasy Mini Kit (Qiagen). To eliminate DNA contamination, we treated the RNA with 2 μL of DNaseI (Invitrogen) at 37°C for 30 min, followed by phenol–chloroform extraction and ethanol precipitation to remove residual DNase. We confirmed the absence of detectable DNA contamination by PCR amplification using pxmF1/pxmR2 primers. For cDNA synthesis, we used SuperscriptIII (Invitrogen) with random primers (Invitrogen), and equal amounts of RNA across all samples. Finally, we performed real-time PCR reactions using SYBER® Premix Dimer Eraser™ (Perfect Real Time) (TaKaRa) on a StepOnePlus™ Real-Time PCR System (Applied Biosystems). Although the expression level of *rpoZ* showed modest variation across conditions, it was minor relative to the expression levels observed for *pxm* and *bapA*. The primer pairs pxmRealF/pxmRealR, bapARealF/bapARealF, and rpoZRealF/rpoZRealR, employed for real-time PCR to amplify the *pxm, bapA*, and *rpoZ* genes, respectively, are listed in Table S2.

### Biofilm formation on polystyrene

To compare biofilm morphologies, we grew biofilms of our test strains on polystyrene (PS) tabs that were propped up against the walls in a 24-well microtiter plate (Iwaki, Shizuoka, Japan). We then carefully filled each well with overnight culture (OD_600_ = 0.05) to partially submerge the tab. The plates were incubated statically overnight at 30°C. Biofilms formed on the PS tabs along the air–liquid interface. To form the PS tabs, we cut Petri dishes into 15 × 10 mm^2^ pieces.

The next day, we performed Live/Dead fluorescent labeling of the biofilms by carefully transferring the tabs to new wells containing PBS with 2.5 μM SYTO9 (Thermo Fischer Scientific, Walthan, MA) and 10 *μ*M propidium iodide (PI) (Thermo Fisher Scientific, Waltham, MA, USA). We incubated the tabs for 15 min at room temperature (RT) and then washed the samples twice by carefully replacing the solution with fresh PBS. We then imaged the fluorescently labeled biofilms using a confocal microscope (LSM880 equipped with a Chameleon Vision laser system, Carl-Zeiss, Oberkochen, Germany) with a water immersion lens (40x; NA0.75). The live cells appear green whereas the dead cells appear magenta [30]. To estimate survival percentage, we subtract the magenta area from the combined green and magenta regions in each confocal slice using MATLAB (Mathworks, Natick MA, USA).

### Microcolony growth in microfluidic chambers

To investigate *P. denitrificans* microcolony formation under different degrees of confinement, we fabricated polydimethylsiloxane (PDMS) microfluidic chambers to limit vertical expansion of microcolonies. We used standard soft lithography techniques [31] to create two-layer microfluidic devices that have deep channels (~80 μm) for media supply and shallow chambers for bacteria imaging [32]. We fabricated two versions of the devices with chamber heights of 2.0 ± 0.1 μm and 1.3 ± 0.1 μm, respectively. We designate microcolony growth in the 2.0 μm chambers “semi-confined” since vertical cell growth is not completely suppressed. In contrast, we designate microcolony growth in the 1.3 μm chambers as “confined” because the cells are restricted to grow in a monolayer. We measured the heights of the different regions in our devices using a confocal profilometer (KEYENCE, Osaka, Japan).

Prior to inoculation into the devices, we removed debris from 8 h liquid culture that could clog the microchambers by filtering the culture with a 5 μm pore syringe filter. We then filled the device with fresh TSB media, inoculated the bacteria through tubing connected to the device using a 1 ml syringe (Terumo, Japan). We then infused media using a syringe pump (Harvard Apparatus, Harvard MA, USA) at a flow rate of 100 μL h^−1^. The devices were incubated at 30°C using a heated microscope stage insert (Tokai hit, Japan). Sequential images were taken on an inverted microscope (Axio Observer.Z1, Carl Zeiss, Germany) with a 100x oil immersion objective (NA1.4).

We tracked cell growth and microcolony area in semi-confined, and 2D confinement chambers by thresholding image sequences and extracting colony areas. To quantify escape events from semi-confined microcolonies, we manually counted cells that escaped from the colony, which we observed to occur exclusively at the colony periphery, in sequential images. To account for colony size differences, escape rates were normalized by the perimeter length of each microcolony.

## Results

### Identification of genes responsible for cellular aggregation in *P. Denitrificans* Pd1222

Shaking liquid cultures of the *P. denitrificans* Pd1222 wild-type (WT) were free of biofilm at the air-liquid interface and lacked sedimented cell aggregates. On solid media, the WT produced smooth colonies. In contrast, liquid cultures of Δ*pdnI* formed both biofilm at the air-liquid interface and millimeter-scale aggregates in liquid culture [26]. On solid media, Δ*pdnI* exhibited a rugose colony morphology (Fig. S1).

To identify the specific gene(s) that regulates aggregation in Δ*pdnI*, we constructed and screened a transposon mutant library for cell aggregation in liquid culture and colony morphology, respectively. Of the ~9000 transposon mutants generated, 23 candidates simultaneously lacked aggregation in liquid culture and demonstrated a smooth colony phenotype on solid media (Fig. S1 and Table S3). Sequencing the transposon insertion site revealed that most insertions occurred between genes Pden_0835 and Pden_0847. Several genes in this region are annotated as glycosyltransferases or polysaccharide biosynthesis proteins (e.g. Pden_0840, Pden_0847), suggesting that this cluster contributes to EPS production. Among these genes, the highest number of transposon insertions were found in Pden_0842. According to the sequence information, Pden_0842 codes for a phosphoglycosyltransferase which belongs to a family of proteins that synthesize cell-surface glycans and glycoconjugates [33–36], that are known to be important for biofilm formation in other bacteria [37]. The impairment of Pden_0842 function due to transposon insertions highlights its role in cellular aggregation in the WT. Since Pden_0842 is a putative polysaccharide biosynthesis gene, we name it *pxm*, to represent the *Paracoccus* eXtracellular matrix synthesis gene.

### Pxm promotes cellular aggregation in Δ*pdnI* mutants

**Figure 1:**
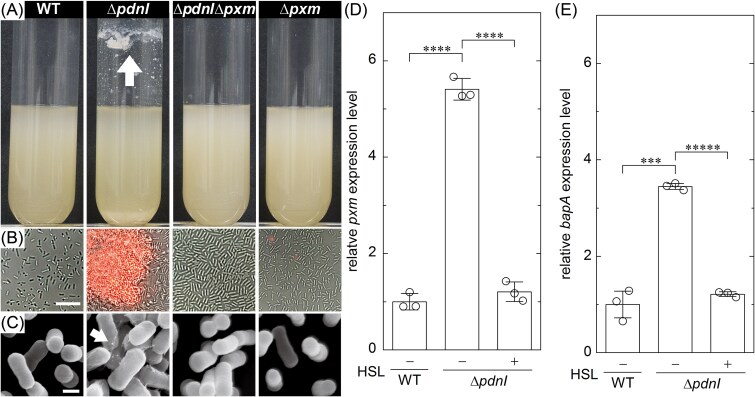
Quorum sensing regulates biosynthesis of the adhesion factors Pxm and BapA. (A) Images of test tube cultures of WT and deletion mutants. The arrow indicates cell aggregation and adhesion to the test tube. Test tube cultures are incubated overnight with shaking at 30°C (see Methods). (B) Bright-field merged with fluorescence images of the different strains incubated with ConA-Alexa594. Scale bar = 10 μm. (C) Scanning electron microscope (SEM) images of cells cultured for 48 h. The white arrow indicates strands of extracellular matrix connecting the cells. Scale bar = 2 μm. (D and E) Normalized expression levels of *pxm* and *bapA* (D) for WT and Δ*pdnI* (E) with (+) and without (−) exogenously added C16-HSL, respectively. We measure the expression levels from RNA i.e. isolated after 16 h of growth. All values are first normalized by the expression level of the constitutive gene, *rpoZ*, and then by the WT expression level to facilitate comparison. We add C16-HSL at a concentration of 5000 nM. The bars represent the mean ± standard deviation (s.d.) (*N* = 3 independent tests). Asterisks indicate statistically significant differences in the means of the labeled groups calculated with Student’s *t*-test (^**^: *P* < .01; ^***^: *P <* .001; ^****^: *P <* .0001).

To test the role of the *pxm* gene in cell aggregation, we constructed single and double knockout mutant strains, Δ*pxm* and Δ*pdnI*Δ*pxm*, respectively. In liquid culture, we found that both Δ*pxm* and Δ*pdnI*Δ*pxm* present phenotypes similar to WT, lacking both biofilm at the air-liquid interface and sedimented aggregates (Fig. 1A). In addition, on solid media they both formed smooth colonies (Fig. S2). These results suggest that Pxm enables cell aggregation and that *pxm* expression is upregulated in Δ*pdnI* relative to WT.

To investigate the Pxm distribution in Δ*pdnl* cellular aggregates, we imaged extracellular polysaccharides (EPS) using both fluorescence and SEM. We labeled the EPS with the fluorescent lectin Concanavalin A (ConA-Alexa), finding that Δ*pdnI* aggregates became brightly labeled, whereas WT, Δ*pdnI*Δ*pxm*, and Δ*pxm,* which lack aggregation, had little or no fluorescence (Fig. 1B). Similarly, SEM revealed the presence of filament-like structures connecting cells from Δ*pdnI* aggregates, whereas these filaments were absent in WT, Δ*pdnI*Δ*pxm*, and Δ*pxm* (Fig. 1C).

Notably, elevated expression of Pxm not only leads to increased aggregation but also appears to enable the biofilm to attach to the glass (see arrow in the tube labeled Δ*pdnI* in Fig. 1A). In *P. denitrificans* Pd1222, the *bapA* gene encodes the cell-surface adhesin, BapA, whose absence results in strains that are unable to attach to surfaces [10]. To investigate the role of BapA in Δ*pdnI* surface adhesion, we constructed the double knockout mutant Δ*pdnI*Δ*bapA*. On solid media, we found that Δ*pdnI*Δ*bapA* had a rough colony phenotype similar to Δ*pdnI* and developed a biofilm in the test tube culture, whereas Δ*bapA* had a smooth colony phenotype and lacked any biofilm in the test tube culture (see Fig. S2). The shared smooth colony phenotype in the nonaggregating strains indicates that BapA plays no significant role in cellular aggregation. Conversely, aggregation and biofilm formation in liquid culture and rough colony phenotype in Δ*pdnI*Δ*bapA* suggests that elevated Pxm biosynthesis promotes cell–cell adhesion and even facilitates cell-surface attachment (Fig. S2).

### Quorum sensing regulates *pxm* and *bapA* expression

Since deletion of *pdnI* led to increased aggregation and surface adhesion, we hypothesized that *pxm* expression is regulated through QS (Figs 1A–C). To quantify *pxm* expression levels in WT and Δ*pdnI* we used reverse transcription quantitative PCR (RT-qPCR), finding ~5-fold higher expression in the Δ*pdnI* mutant than in WT. We additionally measured the resultant effect on *pxm* expression in Δ*pdnI* mutants by supplementing the culture medium with 5000 nM C16-HSL; these cultures had similar expression levels as WT (Fig. 1D). Hereafter we refer to this combination as Δ*pdnI* + C16-HSL. In a similar test, we compared *bapA* expression in WT, Δ*pdnI*, and Δ*pdnI* + C16-HSL, respectively, finding higher expression in Δ*pdnI* relative to WT and Δ*pdnI* + C16-HSL cultures (Fig. 1E). In addition, we found that expression levels of both genes decreased in a C16-HSL concentration-dependent manner (Fig. S3). These results suggest that WT maintains the basal level of secretion of both cell–cell (Pxm) and cell-surface (BapA) adhesion factors through QS.

We investigated the role of the QS signal receptor in Pxm secretion by constructing deletion strains Δ*pdnR* and Δ*pdnRI* as well as strains complemented with *pdnR* (pBpdnR)*, pdnI* (pBpdnI)*,* and *pdnRI* (pBpdnRI); these complemented strains overexpress C16-HSL receptor proteins, C16-HSL synthesis proteins, and both receptor and synthesis proteins simultaneously, respectively. Aggregation was absent in both Δ*pdnR* and Δ*pdnRI* mutants, whereas marginal aggregation was observed in Δ*pdnR*/pBpdnR and extensive aggregation was observed in Δ*pdnRI*/pBpdnR (see columns 5 and 8 in comparison to 6 and 10 in Fig. S4). In both of these complemented strains, supplementing with C16-HSL caused the complete loss of aggregation (see columns 7 and 11 in Fig. S4). The marginal aggregation observed in Δ*pdnR*/pBpdnR suggests that the basal level of expressed *pdnI* was able to generate a sufficient amount of C16-HSL to reduce aggregation. In contrast, Δ*pdnRI*/pBpdnR, which lacks any C16-HSL production, aggregated extensively. The Δ*pdnRI* mutants complemented with *pdnI* also lacked aggregation. Together these results indicate that the presence of PdnR is: (i) necessary for the up-regulation of *pxm* expression; and (ii) that either the loss of PdnR or deactivation through binding with C16-HSL suppresses *pxm* expression.

### The role of Pxm and BapA in biofilm formation

**Figure 2:**
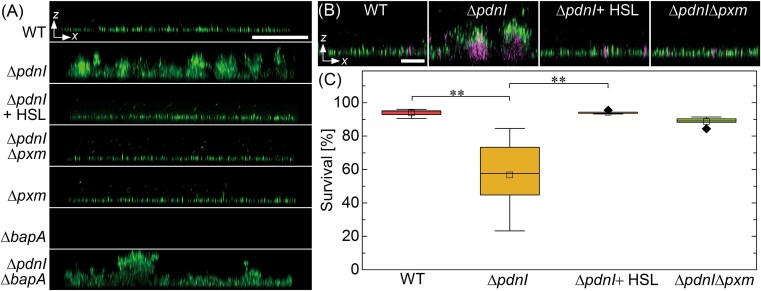
Biofilm morphology in unconfined conditions. (A) Side-view confocal images of WT and mutants grown in an unconfined environment. We culture these strains under static conditions for 48 h, label them with SYTO9, and image them in a polystyrene dish. Scale bar, 50 μm. (B) Representative images stained with SYTO9 and PI. Scale bar, 2 μm. (C) Distribution of survival percentage in WT and mutant strains grown in an unconfined environment. The survival ratios are calculated from the area of the dead cell divided by the area of the live cell. Each condition was tested independently (*N =* 5*)*. The (□) represent the means, the horizontal lines are the medians, and the (◆) represent outliers. Asterisks indicate statistically significant differences in the means of the labeled groups calculated with Student’s *t*-test (^**^: *P <* .01).

The WT is known to form relatively thin biofilms with thicknesses of ~5 μm [10], whereas the Δ*pdnI* mutant forms ~20 μm-thick biofilms [24, 32]. The aggregation phenotypes observed for Δ*pdnI* suggest that the increase in thickness is due to overproduction of Pxm. To investigate its role in biofilm formation, we cultured biofilms of WT and the other mutant strains on rectangular polystyrene tabs. Consistent with previous reports [24, 26, 32], we found thin WT biofilms and thick Δ*pdnI* biofilms. Down regulation or loss of the Pxm synthesis gene resulted in the formation of thin biofilms, as observed for Δ*pdnI* + C16-HSL, Δ*pdnI*Δ*pxm*, and Δ*pxm* (Fig. 2A); thus, confirming our hypothesis that Pxm is involved in thick biofilm formation.

To demonstrate that Pxm is responsible for thick biofilm formation in Pd1222, we constructed overexpression strains by introducing pBpxm, which carries *pxm* under the control of a constitutive promoter. The Pxm overexpression strains WT/pBpxm*,* Δ*pdnI*/pBpxm*,* Δ*pxm*/pBpxm*,* and Δ*pdnI*Δ*pxm*/pBpxm all formed thick biofilms similar to Δ*pdnI* (Fig. S5A). These results confirm that Pxm overproduction is necessary and sufficient for the formation of thick biofilms. Conversely, this indicates that in these conditions the nonaggregating strains express Pxm at a level similar to WT.

Irreversible attachment is the first stage of biofilm formation. In Pd1222, WT uses BapA to attach to surfaces. Disruption of *bapA* ablates biofilm formation since these mutants are unable to attach to surfaces [10]. However, we found that Δ*pdnI*Δ*bapA*, which overexpresses Pxm but lacks BapA, regains the ability to form thick biofilms similar to Δ*pdnI* (Fig. 2A). We note that prior to imaging, we repeatedly washed the cells, which indicates that they were firmly attached to the substrate. Complementing Pxm into BapA-deletion strains enables them to form thick, surface-attached, biofilms (Fig. S5A). These results indicate an additional function of Pxm: overexpression of the polysaccharide plays a secondary role in mediating biofilm surface attachment.

### Increased cell death observed in thick biofilms

The rate of cell death is known to increase inside thick biofilms due to competition for essential nutrients [38]. To determine if Pxm overexpression can lead to increased cell death, we used live/dead fluorescent cell staining on thin and thick biofilms. From our confocal images, we observed relatively few dead cells in the thin biofilms formed by WT, Δ*pdnI* + C16-HSL, and Δ*pdnI*Δ*pxm*, whereas inside the thick Δ*pdnI* biofilms, we found a mass of dead cells, which appear magenta in Fig. 2B. From these confocal images, we estimated the survival percentage by measuring the volume of dead cells within the biofilm. In the thin biofilms, ~90% of the cells are alive, whereas in the thick Δ*pdnI* biofilms, ~60% of the cells are alive (Fig. 2C).

### Role of Pxm and BapA in microcolonies in semi-confined environments

**Figure 3:**
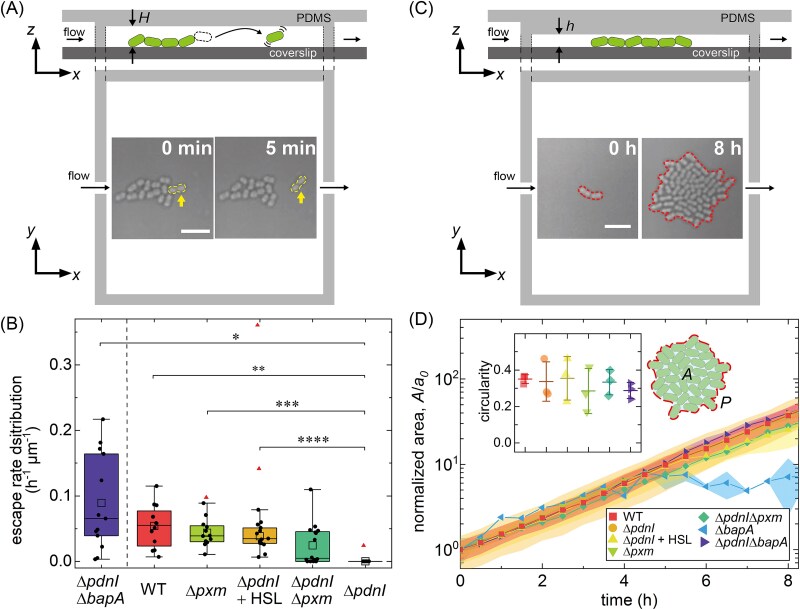
Colony morphology in semi-confined and confined environments. (A) Schematic of the “semi-confined” microfluidic chamber geometry. (upper) Side view of the chamber depicting a cell escaping from the microcolony. The dashed lines represent vertical walls that have openings through which media flows. (lower) Top view of the chamber with an image sequence capturing cell escape, indicated by the arrow. The height of the chamber, *H*, is ~2 μm. Scale bar, 5 μm. (B) Distribution of escape rates from semi-confined microcolonies. For each strain, the number of tracked colonies is *n* ≥ 12 for *N* ≥ 3 independent tests. In each box, the (•) represent measurements, the horizontal line represents the median, (□) represent the means, and the (▲) represent outliers. The mean values are compared using Welch’s *t*-test corrected with the Bonferroni-Holm method. Asterisks indicate statistically significant differences in the means (^*^: *P <* .05; ^**^: *P <* 0.01; ^***^: *P <* .001). The mutant Δ*bapA* is omitted because it is unable to attach to the chambers. Unlike the other tested strains, Δ*pdnI*Δ*bapA* micro-colonies grew from small aggregates and no single cells, indicated by the vertical dashed line. (C) Schematic of “confined” microfluidic chamber geometry. (upper) Side view showing a growing microcolony. (lower) Top view of the chamber with an inset image sequence showing microcolony growth after ~6 divisions, corresponding to ~64 cells. The height of this chamber, *h*, is ~1.3 μm. Scale bar, 5 μm. (D) Normalized colony area, *A/a*_0_, plotted as a function of time for the different strains. Here, *A* is the colony area and *a*_0_ is the initial colony area (area of a single cell). The symbol and filled regions represent the mean ± s.d. (*N* ≥ 3). (Inset) Microcolony circularity after ~6 divisions (~9 h), excluding Δ*bapA*. The schematic shows the colony area and perimeter, *P*, of a microcolony. Microcolony circularity is given by 4π*A/P*^2^. The symbols represent experimental data, the wide and narrow horizontal lines represent the means and s.d., respectively. There are no significant differences between the means for all samples calculated with the Student t-test.

BapA and Pxm both influence *P. denitrificans* biofilm morphology and development in unconfined conditions (Fig. 2). We investigated how their roles may change during microcolony development in an environment with confinement such as the soil. Here, we cultured different strains under semi-confinement in our 2 μm-tall microfluidic chambers. *P. denitrificans* are 0.8 ± 0.1 μm in diameter and 1.7 ± 0.3 μm in length. During microcolony expansion in this environment, we hypothesize that peripheral cells may detach from the chamber surface while remaining connected to the colony (Fig. 3A). In this scenario, if the intercellular connection is lost, the cell escapes and diffuses away (see image sequence in Fig. 3A). The low ceiling height of the chamber facilitates the enumeration of the cell-escape events from microcolonies while reducing invasion and interference from planktonic cells.

From time-lapse sequences, we measured the number of cells that escape from a colony during colony growth for the strains shown in Fig. 2A. We normalized these rates by the instantaneous microcolony perimeter to account for colony size differences, calling this value the escape rate. We note that Δ*bapA* was unable to form microcolonies in these devices. We found that all strains except Δ*pdnI* demonstrated cell escape at a nonzero rate, with WT, Δ*pxm*, and Δ*pdnI* + C16-HSL having a median escape rate of ~0.05 cells h^−1^ μm^−1^ (Fig. 3B). We note that this cohort forms thin biofilms, expresses basal levels of BapA, and either has low or zero Pxm. Of all thin-biofilm forming strains (Fig. 2A), the Δ*pdnI*Δ*pxm* mutant, which overproduces BapA, had the lowest median escape rate; however, its escape rates are bimodally distributed, as determined with Hartigan’s test for multimodality (*p*-value = 0.02). The higher rates cluster near 0.05 cells h^−1^ μm^−1^, like the other thin-biofilm formers (WT, Δ*pxm*, and Δ*pdnI* + C16-HSL), while the lower escape rates cluster near zero, like Δ*pdnI* (Fig. 3B).

In contrast, the Δ*pdnI*Δ*bapA* mutant, which also forms thick biofilms, had the widest distribution of escape rates with a median i.e. similar to, but trending higher, than WT (Fig. 3B and Movie S1). We note that unlike the other strains tested, Δ*pdnI*Δ*bapA* microcolonies begin from small clusters and not individual cells. All BapA expressing strains can attach to surfaces as single cells; however, due to the lack of BapA in Δ*pdnI*Δ*bapA*, single cells are unable to effect surface attachment inside the chamber. Instead, our assay selects small cell clusters that can attach in a manner similar to unconfined conditions (Fig. 2A).

### Colony expansion under 2D confinement unaffected by Pxm overexpression

To further investigate the role of adhesion factors on microcolony formation in highly confined conditions, we cultured microcolonies in ~1.3 μm-tall microfluidic chambers (Fig. 3C) [32]. We inoculated a dilute cell suspension to seed the formation of monoclonal colonies in the chambers and tracked colony expansion for ~8 h (~6 division cycles) (Movie S2). Under these conditions, as in the semi-confined conditions, cell escape from all microcolonies occurred. However, due to the close proximity of the ceiling and floor, cells frequently reattached very close to the mother colony, making analysis difficult. Instead we focused on microcolony development and structure.

Under high confinement, all strains other than Δ*bapA* formed colonies that expanded exponentially, as shown in Fig. 3D and Fig. S6. At the end of each test, we measured the colony circularity, 4π*A/P*^2^, where *A* and *P* are colony area and perimeter, respectively, finding similar values for all strains (Fig. 3D, inset). We note that unlike in semi-confinement, despite lacking BapA, the Δ*pdnI*Δ*bapA* colony morphology was similar to the morphology presented by BapA-expressing strains. Conversely, the Δ*bapA* mutant, which lacks BapA and expresses Pxm at a low level, mainly diffuses around the chamber as single cells. Occasionally a small cluster of 2–4 cells appeared to become wedged between the ceiling and floor, which could be the result of small inhomogeneities in the chamber height.

## Discussion

The ability of bacteria to form surface-associated communities encased in an EPS matrix is a widespread strategy, involving a complex interplay of adhesins and matrix components. Much of this process has been clarified using the motile, model organisms *V. cholerae* and *P. aeruginosa* [39–42]. Much less is known about the mechanisms of biofilm formation in nonmotile bacteria. Our study examines biofilm formation in the nonmotile *P. denitrificans*, which is relevant to environments that experience dynamic changes in hydration, which are linked to changes in oxygen and other nutrients levels [43].

Our findings link the *pdnI/R* QS system to the regulation of two key adhesion factors, the hypothetical polysaccharide biosynthase Pxm and BapA (Fig. 1, S2). In Δ*pdnI*, the absence of the QS signal C16-HSL leads to accumulation of Pxm and aggregation. This behavior is reversed with exogenous C16-HSL. Similarly, QS regulates BapA, with higher levels in Δ*pdnI* correlating with slightly enhanced surface attachment (Fig. 3B). The contrasting smooth/thin phenotypes (WT) and rugose/thick (Δ*pdnI*) biofilms highlight the importance of QS in balancing these factors (Fig. 2A).

QS-mediated downregulation of *pxm* in WT is important for limiting biofilm thickness; this likely provides a selective advantage in nutrient-limited and porous environments, where thick biofilms choke-off nutrient flow. The necrotic cores in thick Δ*pdnI* biofilms support the idea that thick biofilms can deprive biofilm cells of nutrients, causing cell death (Fig. 2B and C). The ability of WT to form thin biofilms minimizes nutrient gradients across the biofilm, thus promoting cell viability in porous media, such as in soils [44]. Although active motility may lessen the effects of clogging, they represent a significant challenge for nonmotile bacteria, which must rely on diffusion and flow for dispersal in spatially constrained habitats [44, 45].

To clarify the roles of BapA and Pxm in colony cohesion, we measured single-cell dispersal from microcolonies inhabiting a model porous environment, using microfluidics. The similar escape rates observed in WT, Δ*pxm*, and Δ*pdnI* + C16-HSL—all exhibiting low Pxm and moderate BapA levels – suggest that surface attachment mediated by BapA is a primary determinant of microcolony cohesion under flow (Fig. 3B). The lack of cell escape in Δ*pdnI* microcolonies, where both BapA and Pxm are overexpressed, suggests a synergy between strong cell-surface and cell–cell interactions.

Double knock-out mutants (Δ*pdnI*Δ*pxm* and Δ*pdnI*Δ*bapA*) revealed that while BapA is the dominant factor in initial surface anchoring, overexpressed Pxm contributes a secondary level of adhesion. Surprisingly, whereas Pxm overexpression leads to thick biofilms when unconfined (Fig. 2A), in these conditions, Pxm overexpression alone is insufficient to halt escape through strong cell–cell adhesion (see Δ*pdnI*Δ*bapA* in Fig. 3B). In contrast, the bimodal escape behavior in Δ*pdnI*Δ*pxm* further highlights the importance of BapA overexpression. We conclude that cells escape when they grow away from the surface, where BapA functions (Fig. 3A).

Under even greater confinement (Fig. 3C and D), we observed the convergence of colony morphology in all strains except Δ*bapA*. Previous work has shown that larger surface adhesion forces lead to rounder microcolonies in 2D [46]. The similar colony circularity observed in our experiments suggests that the additional surface area for adhesion due to the proximity of both ceiling and floor, minimizes phenotypic differences that would arise from varying levels of BapA and Pxm.

**Figure 4:**
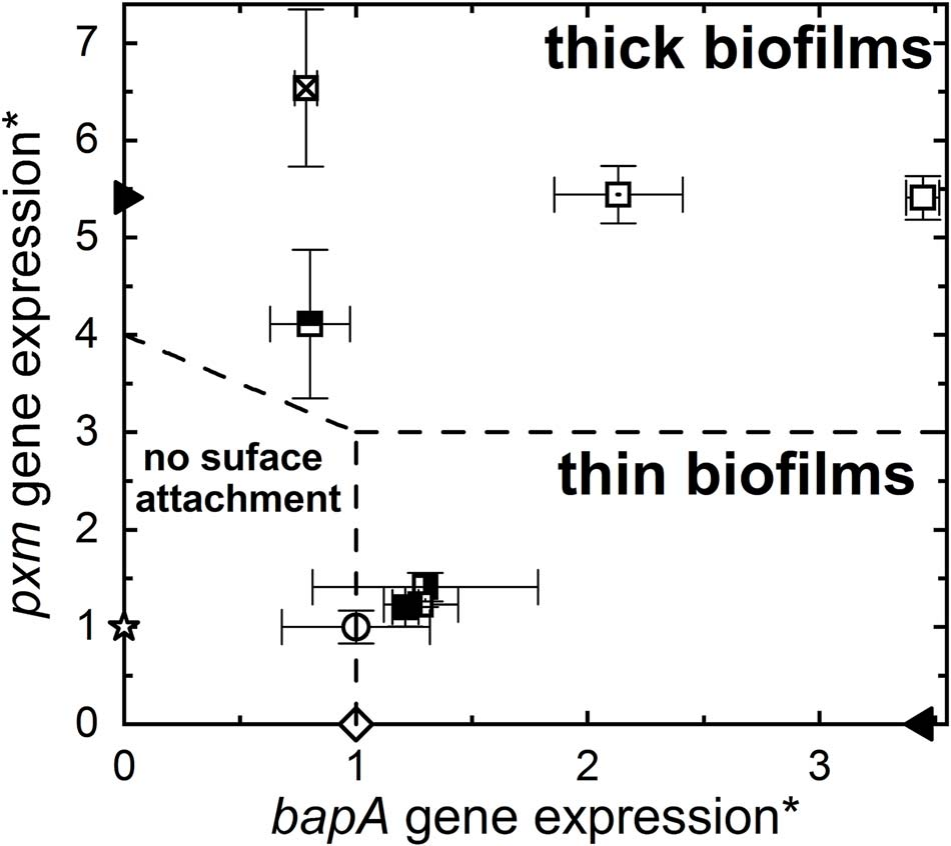
Phase diagram of *P. Denitrificans* biofilm morphology formed from normalized *bapA* and *pxm* expression levels. The asterisk in the axis titles indicates that *bapA* and *pxm* gene expression levels are normalized to those of the WT (see Fig. 1D and E and Fig. S3). The dashed lines indicate the approximate boundaries between regions. The symbols for each strain are as follows: (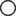) WT; Δ*pdnI* with (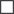) 0, (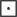) 0.05, (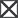) 0.5, (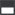) 5, (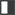) 50, (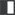) 500, (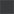) 5000 nM C16-HSL added to the culture medium, respectively; (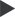) Δ*pdnI*Δ*bapA*; (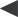) Δ*pdnI*Δ*pxm*; (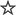) Δ*bap*; (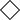) Δ*pxm*. Symbols with error bars represent direct measurements of gene expression. We assume the expression levels of *bapA* in Δ*pxm*, and *pxm* in Δ*bapA* are the same as in WT. Similarly, we assume the expression levels of *bapA* in Δ*pdnI*Δ*pxm*, and *pxm* in Δ*pdnI*Δ*bapA* are the same as in Δ*pdnI*.

The two-component adhesion system employed by *P. denitrificans* makes it a valuable model for biofilm development in a “minimal” system, contrasting with the complex mechanisms used by *P. aeruginosa* and *V. cholerae*. We construct a semi-quantitative phase diagram that illustrates the *bapA* and *pxm* expression-level dependent biofilm architecture for each strain tested (Fig. 4). The preference for thin biofilms likely represents an adaptation to porous environments, mitigating the risk of nutrient flow obstruction [47] and possibly compensating for the inability to actively move. For nonmotile organisms, communication may be critical to survival by regulating behavior. Environmental confinement influences not only cell dispersal but may also lead to the local accumulation of QS signals. In such an environment, the *P. denitrificans* QS system ensures suppression of any aggregative phenotypes that may appear. This adaptation to confinement may preclude the ability of *P. denitrificans* to form thick biofilms when unconfined.

It is an open question how *P. denitrificans* transmits the hydrophobic C16-HSL QS signal molecules: are they freely diffusible in the aqueous environment; are they packaged in amphiphilic structures [48, 49] like membrane vesicles; and/or are they transmitted physically through cell-to-cell contact? This question warrants further investigation to fully understand the coordination of multicellular behavior in complex microenvironments that may reveal strategies for modulating biofilm development in ecological and engineered systems.

## Supplementary Material

251027_pd_pxm_SI_FIN_ycaf198

## Data Availability

The datasets and analysis code supporting this study are archived in Figshare (https://doi.org/10.6084/m9.figshare.29376734). The raw image data underlying this study are available from the corresponding authors upon request.
