# Supplementary material for: Quorum sensing regulates a polysaccharide biosynthesis gene to control cell aggregation in Paracoccus denitrificans
Source: ISME Commun. 2025 Oct 31;6(1):ycaf198. doi: 10.1093/ismeco/ycaf198 (PMC13235738; doi:10.1093/ismeco/ycaf198)
Supplement: 251027_pd_pxm_SI_FIN_ycaf198 [file 251027_pd_pxm_si_fin_ycaf198.docx]

# Supplementary Information

Quorum sensing regulates a polysaccharide biosynthesis gene to control cell aggregation in *Paracoccus denitrificans*

Kana Morinaga^1✝^, Kohei Takahashi^1✝^, Ryo Nagasawa^1^, Wenzhi Tao^1^, Shinya Sugimoto^2^, Nozomu Obana^3,4^, Nobuhiko Nomura^4,5,6^, Andrew S. Utada^4,5,6*^, and Masanori Toyofuku^4,5,6*^

^1^Graduate School of Life and Environmental Sciences, University of Tsukuba, 1-1-1 Tennodai, Tsukuba, Ibaraki 305–8572, Japan

^2^Department of Bacteriology and Jikei Center for Biofilm Research and Technology, The Jikei University School of Medicine, 3-25-8, Nishi-Shimbashi, Minato-ku, Tokyo, 105-8461, Japan.

^3^Transborder Medical Research Center, Faculty of Medicine, University of Tsukuba, 1-1-1 Tennodai, Tsukuba, Ibaraki 305–8577, Japan

^4^Microbiology Research Center for Sustainability (MiCS), University of Tsukuba, 1-1-1 Tennodai, Tsukuba, Ibaraki 305–8572, Japan

^5^Institute of Life and Environmental Sciences, University of Tsukuba, 1-1-1 Tennodai, Tsukuba, Ibaraki 305–8572, Japan

^6^Tsukuba Institute for Advanced Research, University of Tsukuba, 1-1-1, Tennodai, Tsukuba, Ibaraki, 305-8577, Japan

^✝^Indicates equal contribution

**Corresponding Authors**:

Andrew S. Utada

Masanori Toyofuku

**This file includes**:

Extended Materials and Methods

Tables S1-S3

Figures S1-S6

Supplementary References

**Extended Materials and Methods**

**LuxI/LuxR homologue naming convention**

In *P. denitrificans*, different naming conventions have been used for the *luxI* (PdnI) and *luxR* (PdeR) homologues [[1–3]](https://sciwheel.com/work/citation?ids=14669134,14672529,17840018&pre=&pre=&pre=&suf=&suf=&suf=&sa=0,0,0). In this report, we refer to the *luxI* and *luxR* homologues as PdnI and PdnR, following the naming convention of the earlier report.

**Random transposon mutagenesis and screening**

To construct a transposon library of Δ*pdnI* mutants, we used Tn5-carrying plasmid pSUP2021 as previously described [[4–6]](https://sciwheel.com/work/citation?ids=2068593,14650228,10111375&pre=&pre=&pre=&suf=&suf=&suf=&sa=0,0,0&dbf=0&dbf=0&dbf=0). We conjugated the plasmid from *E. coli* S17-1 to *P. denitrificans* Δ*pdnI* on a membrane filter, and the transposon mutants were spread on LB selection plates supplemented with 100 μg ml^-1^ ampicillin and incubated for 48 h. During the first screening, we selected mutant strains that showed a smooth colony phenotype. We carried out a second screening to further select mutants that lost aggregation in shaking liquid culture (190 rpm, 30º C). We found 23 candidates that did not aggregate in the test tube cultures. To identify the insertion sites, we sequenced DNA fragments that were amplified by arbitrary PCR [[7]](https://sciwheel.com/work/citation?ids=229693&pre=&suf=&sa=0).

**Construction of deletion mutants**

We constructed gene deletion mutants using homologous recombination, as following the procedure previously described [[1]](https://sciwheel.com/work/citation?ids=14669134&pre=&suf=&sa=0). To generate the Δ*pdnR* mutant, we first amplified flanking regions of *pdnR* using the primer pairs luxRF1/luxRR1 and luxRF2/luxRR2. These PCR fragments were fused by overlap extension PCR using the primers luxRF0/luxRR0. The resulting amplicon was digested with PstI and XbaI, and ligated into the mobilization vector pK18mobsacB [[8]](https://sciwheel.com/work/citation?ids=4217473&pre=&suf=&sa=0). The resultant vector was named pK18-Pdn-pdnR, and was used for homologous recombination to delete *pdnR.* The gene deletion vector was delivered into *P. denitrificans* Pd1222 by conjugation using *E. coli* S17-1 [[4, 9]](https://sciwheel.com/work/citation?ids=4941718,2068593&pre=&pre=&suf=&suf=&sa=0,0).

Following the same procedure, we generated the Δ*pdnRI* and Δ*pxm* mutants. To generate Δ*pdnRI* we used pK18-Pdn-pdnRI. The construction of pK18-Pdn-pdnRI was proceeded as follows: PCR primers, luxIRF1/luxIRR1 and luxIRF2/luxIR2 were used to amplify flanking regions of the *pdnRI* operon. The PCR fragments were fused by overlap extension PCR using luxRF0/luxIR0 primers. The resulting PCR amplicon was digested with *PstI* and *SpeI* before being cloned into pK18mobsacB resulting in the construction of pK18-Pdn-pdnR. To generate Δ*pxm* we used pK18-Pdn-pxm. The construction of pK18-Pdn-pxmwas proceeded as follows: PCR primers pxmF1/pxmR1 and pxmF2/pxmR2 were used to amplify flanking regions of *pxm.* The PCR fragments were fused by overlap extension PCR using pxmF0/pxmR0 primers. The resulting PCR amplicon was digested with *PstI* and *XbaI* before being cloned into pK18mobsacB resulting in the construction of pK18-Pdn-pxm.

We also generated double mutants Δ*pdnI*Δ*pxm* and Δ*pdnI*Δ*bapA*, using pK18-Pdn-pxm, and pK18-Pdn-luxI vectors [[1]](https://sciwheel.com/work/citation?ids=14669134&pre=&suf=&sa=0). *pxm* was deleted from Δ*pdnI* using pK18-Pdn-pxm to generate Δ*pdnI*Δ*pxm*, while *pdnI* was deleted, using pK18-Pdn-luxI, from Δ*bapA* [[10]](https://sciwheel.com/work/citation?ids=14650241&pre=&suf=&sa=0&dbf=0) to generated Δ*pdnI*Δ*bapA*.

**Construction of vectors for complemented strains**

To construct pBpdnI used for the complementation *pdnI*, we used the primers luxIcompF/luxIcompR to amplify *pdnI*. luxIcompF encodes a SD sequence taken from GAPDH (Pden_4465) before the start codon of *pdnI*. Similarly, to construct pBpdnR used for the complementation *pdnR,* luxRcompF/luxRcompR primers were used. This primer pair amplifies *pdnR* including its putative SD sequence. pBpdnRI used for the complementation *pdnR and pdnI* was constructed using luxRcompF/luxIcompR primers. Each PCR product was cloned into the multicloning site of pBBR1MCS-2 [[11]](https://sciwheel.com/work/citation?ids=2068591&pre=&suf=&sa=0&dbf=0), and the resulting vectors (pBpdnI, pBpdnR, pBpdnRI) were replicated in *E. coli* DH5α. The vectors were delivered into *P. denitrificans* Pd1222 by conjugation using *E. coli* S17-1.

To construct pBpxm for *pxm* complemented strains, we used the primers pxmcompF/pxmcompR to amplify a 1,004 bp fragment, which include *pxm* ORF and a putative *pxm* promoter region. We used the primers T0TermF/T0TermR to clone a bacteriophage lambda T0 terminator from pPROBE-*gfp*[ASV]. We digested pBBPML with *XbaI* and *KpnI* and ligated it with two PCR amplicons (the region of *pxm* and T0 terminator) using HiFi DNA Assembly Master Mix. pBpxm was delivered into *P. denitrificans* Pd1222 by conjugation using *E. coli* S17-1.

**Growth Curve Measurement**

Frozen cultures were streaked on LB agar plates and incubated at 30 °C for 36 hours. 4~5 colonies were then inoculated into TSB liquid medium and cultured overnight at 30 °C with continuous shaking at 190 rpm. The overnight culture was sub-cultured in fresh TSB medium at a 1:100 ratio and grown for 6-8 hours to reach the logarithmic phase. This log-phase culture served as the inoculum for the main experiment. The main cultures were grown in 500 mL Erlenmeyer flasks containing 100 mL of TSB medium, set up in triplicate. Cultures were inoculated to a starting optical density at 600 nm (OD_600_) of 0.01 and then incubated at 30 °C with shaking at 190 rpm. At predefined time points (0, 2, 3, 4, 5, 6, 7, 8, 9, 10, 11, 12, 13, 14, 16, and 24 h), samples were withdrawn to measure the OD_600_ using a spectrophotometer and disposable plastic cuvettes. For readings below 0.8, the absorbance was measured directly. For readings of 0.8 and above, samples were diluted 1/10 in fresh TSB medium, vortexed vigorously, and the absorbance of the diluted sample was measured. The final reading was then calculated by multiplying the result by 10. The entire experiment was performed three times as independent biological replicates (N=3). Growth curves were plotted using the mean values and standard deviations from the biological replicates.

## Supplementary Table S1. List of strains and plasmids used in this study.

| Strain, plasmid | Relevant characteristics | Source or reference |
| --- | --- | --- |
| **Strains** |  |  |
| *Paracoccus denitrificans* |  |  |
| Pd1222 | Wild type, Rif^r^ | de Vries et al, (1989) |
| Δ*pdnI* | Pd1222 mutant with a deletion in the *pdnI* gene | Toyofuku et al, (2017) |
| Δ*pdnR* | Pd1222 mutant with a deletion in the *pdnR* gene | This study |
| Δ*pdnRI* | Pd1222 mutant with a deletion in the *pdnR* and the *pdnI* gene | This study |
| Δ*pxm* | Pd1222 mutant with a deletion in the *pxm* gene | This study |
| Δ*bapA* | Pd1222 mutant with a deletion in the *bapA* gene | Yoshida et al, (2017) |
| Δ*pdnI*Δ*pxm* | Pd1222 mutant with a deletion in the *pdnI* and the *pxm* gene | This study |
| Δ*pdnI*Δ*bapA* | Pd1222 mutant with a deletion in the *pdnI* and the *bapA* gene | This study |
|  |  |  |
| *Escherichia coli* |  |  |
| DH5α | *E. coli* strain for transformation (F^−^ , *lacZ* M1, *recA*) | TaKaRa |
| S17-1 | Mobilizer strain for conjugation | Simon et al, (1986) |
|  |  |  |
| **Plasmids** |  |  |
| pK18 mobsacB | Allelic exchange suicide vector, Km^r^ | Schäfer et al, (1994) |
| pK18-Pdn-luxI | *pdnI* deletion cassette in pK18mobsacB | Toyofuku et al, (2017) |
| pK18-Pdn-pdnR | *pdnR* deletion cassette in pK18mobsacB | This study |
| pK18-Pdn-pdnRI | *pdnR* and *pdnI* deletion cassette in pK18mobsacB | This study |
| pK18-Pdn-pxm | *pxm* deletion cassette in pK18mobsacB | This study |
| pBBR1MCS-2 | Broad-host-range vector, Km^r^ | Kovach et al, (1995) |
| pBpdnI | pBBRMCS-2 carrying *pdnI* fused to the SD sequence of GAPDH (Pden_4465) | This study |
| pBpdnR | pBBRMCS-2 carrying *pdnR* gene | This study |
| pBpdnRI | pBBRMCS-2 carrying *pdnR* and *pdnI* gene | This study |
| pBpxm | pBBPML carrying *pxm* gene | This study |
| pSUP2021 | Transposition vector; Tn5 | Simon et al, (1986) |

## Supplementary Table S2. List of primers and their sequences used in this study.

| Primers | Sequence 5 '→ 3' | Source or reference |
| --- | --- | --- |
| luxRF1 | GACTATGACAAGCTGACCATGAAGGTC | This study |
| luxRR1 | CCGTATTACAGCAAGCGGTACGCGCGAGACGACATGACTT | This study |
| luxRF2 | AAGTCATGTCGTCTCGCGCGTACCGCTTGCTGTAATACGG | This study |
| luxRR2 | GTCCAGCTTGTCCTTGACCTTCCAG | This study |
| luxRF0 | AACTGCAGCATCATCCAGGACCAGCTTT | This study |
| luxRR0 | GCTCTAGATGTTGAAGCGGTGGATCTCATC | This study |
| luxIRR1 | GCCCATCAGTGCATCTTGGCCGCGCGAGACGACATGACTT | This study |
| luxIRF2 | AAGTCATGTCGTCTCGCGCGGCCAAGATGCACTGATGGGC | This study |
| luxIF2 | TTCCCACAGGGTCATGGTGTCCAG | Toyofuku et al, (2017) |
| luxIR2 | GGACTAGTGCCAGGATATTGACCCCATA | Toyofuku et al, (2017) |
| pxmF1 | GCTTTTGTCGGGGATCATGC | This study |
| pxmR1 | ACCGCTCAGTAGCCGGTGCACGTGGTGTGCCGCATTTGAA | This study |
| pxmF2 | TTCAAATGCGGCACACCACGTGCACCGGCTACTGAGCGGT | This study |
| pxmR2 | GCGATGTTCAAGGCGCAATA | This study |
| pxmF0 | AACTGCAGGTCGACGAGCCCTATGAGTG | This study |
| pxmR0 | GCTCTAGATGGCTTCCATGATGACCACC | This study |
| luxIcompF | ACGGTACCCGGCGGAGGAAACCTATGCAGACCACCACACTTTC | This study |
| luxIcompR | GGACTAGTTCAGTGCATCTTGGCCGCCA | This study |
| luxRcompF | ACGGTACCTCCTGGTAACCCGAAGAAA | This study |
| luxRcompR | GGACTAGTTTACAGCAAGCGGTAATCCT | This study |
| pxmcompF | CAAAAGCTGGGTACCATGAACAACCCTCGATTGCG | This study |
| pxmcompR | CTAATTAAGCTCAGTAGCCGGTGCAGCGCA | This study |
| T0TermF | CGGCTACTGAGCTTAATTAGCTGAGCTTGG | This study |
| T0TermR | TGGCGGCCGCTCTAGAGCTAGCTTGGATTCTCACCA | This study |
| pxmRealF | CGGCCGATGATGATCGA | This study |
| pxmRealR | CGTTGCGATCCGAGATCTG | This study |
| bapARealF | CAGTACGGTTCGCCTTCCAT | This study |
| bapARealR | CCGAATGATCTCACCCGAGTT | This study |
| rpoZRealF | TCGCGACAACGACAAGAATC | This study |
| rpoZRealR | CTGCTGGGCCTCGATCAT | This study |

*restriction enzyme sites are underlined

##

## Supplementary Table S3. List of transposon insertion sites in Δ*pdnI*.

| Gene number | Definition | Hypothetical function | Strain No. |
| --- | --- | --- | --- |
| Pden_0073 | cobyrinic acid a,c-diamide synthase | metabolism | Tn5 |
| Pden_0785 | 50S ribosomal protein L17 | translation | Tn13 |
| Pden_0786 | response regulator receiver protein (*pdnR*) | regulation protein | Tn9, Tn19 |
| Pden_0835 | hypothetical protein | - | Tn15, Tn16, Tn20, Tn23 |
| Pden_0838 | amino acid adenylation protein | amino acid modification | Tn1, Tn2, Tn6, Tn10 |
| Pden_0839 | 4’-phosphopantetheinyl transferase | fatty acid biosynthesis | Tn11 |
| Pden_0842 | undecaprenyl-phosphate galactose phosphotransferase | polysaccharide biosynthesis | Tn4, Tn7,　Tn8, Tn14, Tn17, Tn18 |
| Pden_0847 | polysaccharide biosynthesis protein | polysaccharide biosynthesis | Tn12 |
| Pden_1293 | serine acetyltransferase CysE | amino acid biosynthesis | Tn3 |
| Pden_2297 | UTP-glucose-1-phosphate uridylyltransferase | Polysaccharide biosynthesis | Tn21 |
| Pden_4037 | D-isomer specific 2-hydroxyacid dehydrogenase | Polysaccharide biosynthesis | Tn22 |


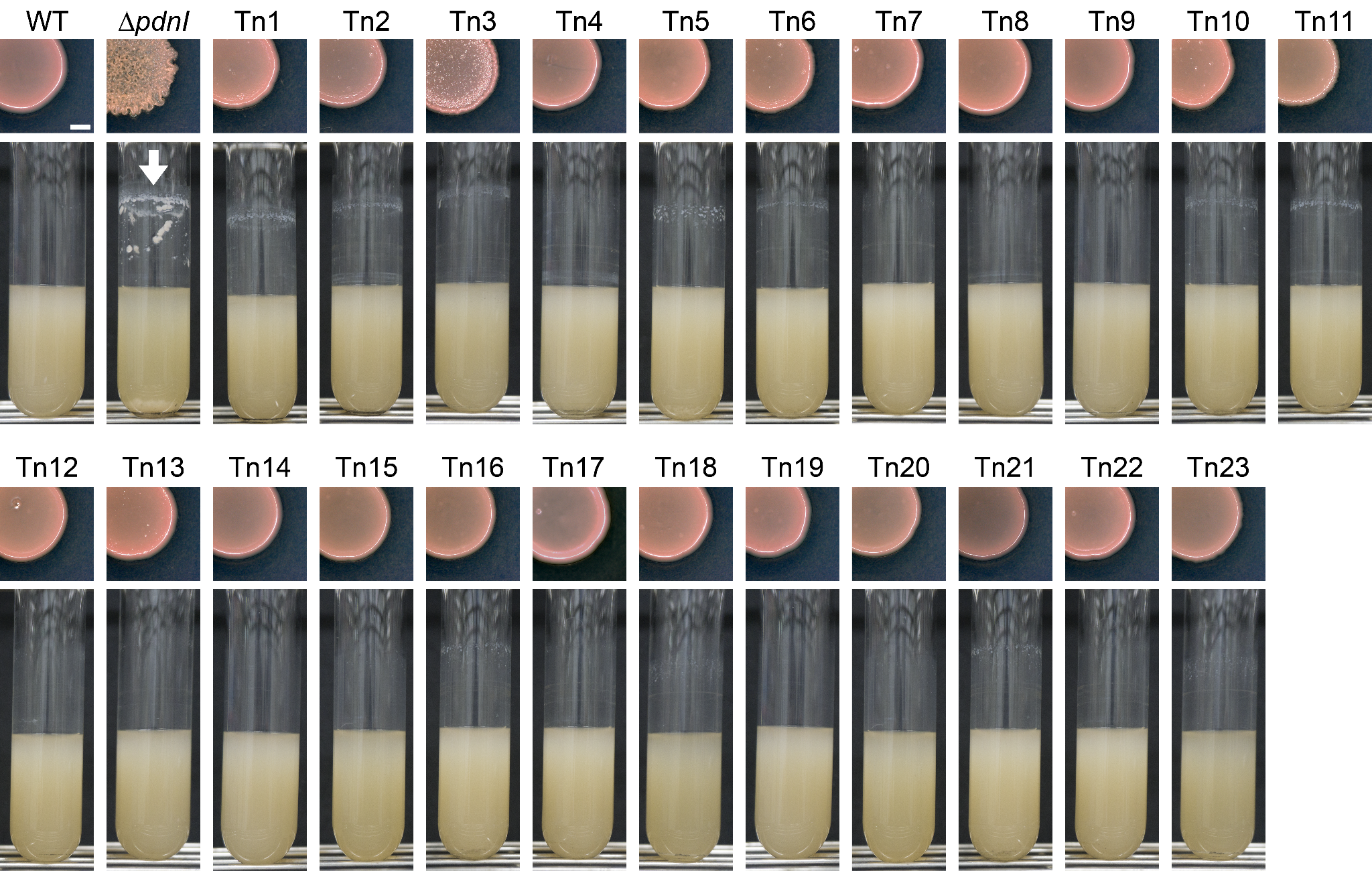


## Supplementary Figure S1. Screening of transposon mutants in the Δ*pdnI* background that lack aggregation. After mutagenesis, the mutant strains were cultured on LB agar for 48 h. Images of the respective mutant are shown above each test tube. The scale bar in the WT image is 2 mm and applies to all colonies. After selection on LB plates, we tested the aggregation phenotype in liquid culture. These cultures were incubated overnight with shaking at 30º C. These images are representative of biologically independent experiments (N=3).

##
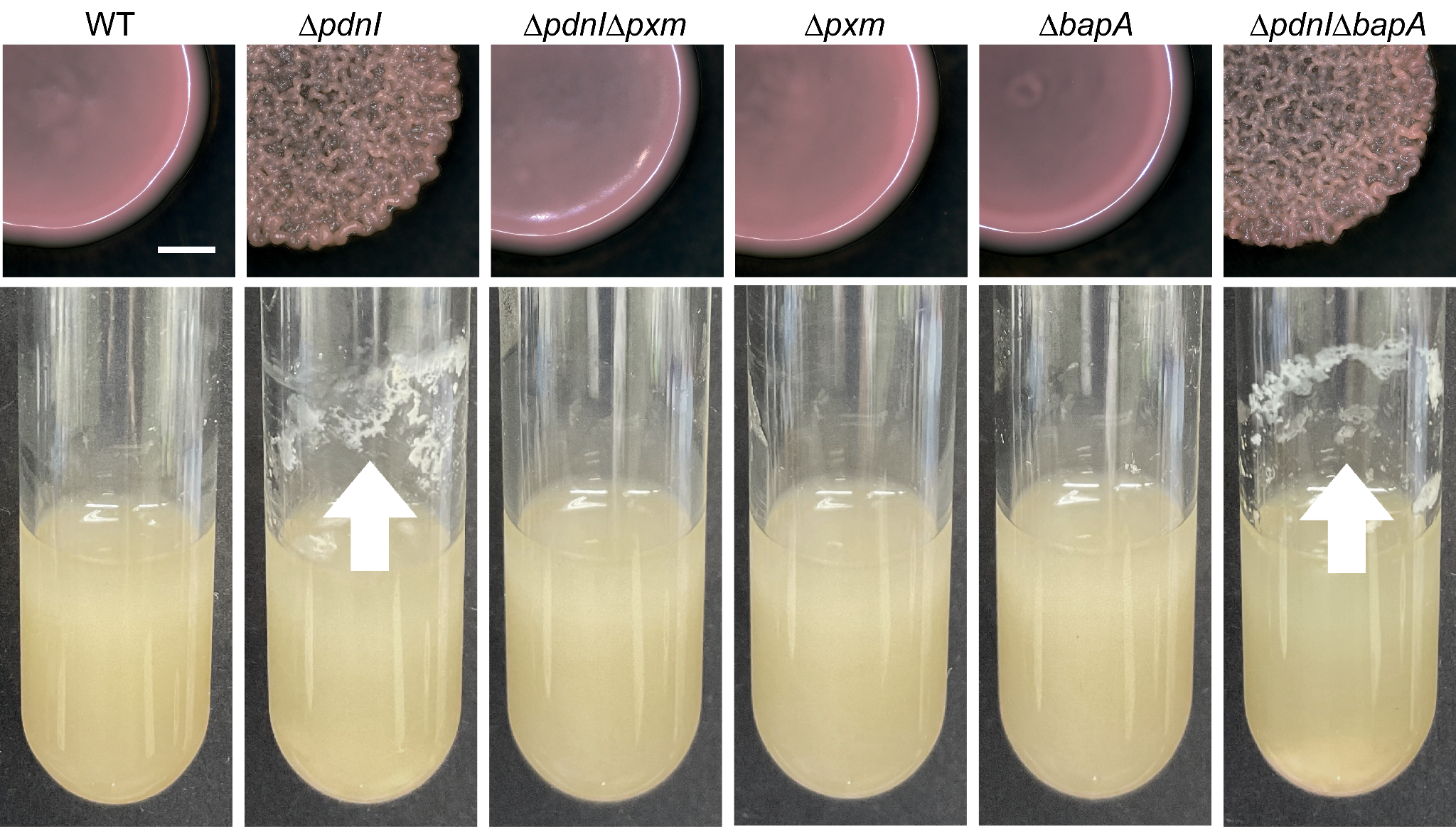
Supplementary Figure S2. Solid and liquid culture aggregation phenotypes for WT and deletion mutants. LB agar plates were incubated for 48 h at 30 ºC, while liquid cultures were incubated overnight with shaking at 30 ºC. The scale bar in the WT image is 2 mm and applies to all colonies. The arrows in the test tube images indicate cell aggregation and adhesion to the glass wall. These images are representative of biologically independent experiments (N=3).

##
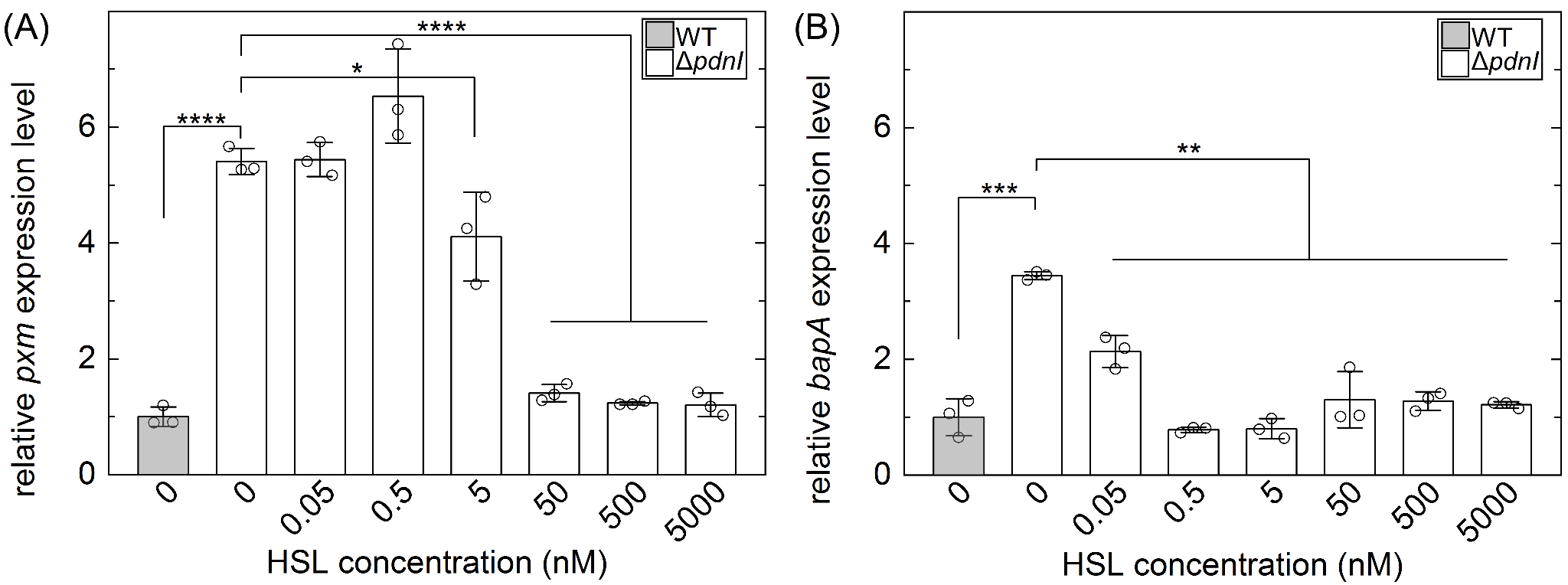
Supplementary Figure S3. Real-time PCR expression levels of *pxm* and *bapA*. The expression levels of (A) *pxm* and (B) *bapA* in WT and Δ*pdnI*, respectively, are shown for different concentrations of exogenously added C16-HSL. We measured expression levels from RNA that was isolated from these strains after 16 h of growth. All values were first normalized by the WT expression level of the constitutive gene *rpoZ* to facilitate comparison. The bars and error bars represent the mean ± standard deviation (s.d.) (N=3). Asterisks indicate statistically significant differences in the means of the labeled groups calculated with Student’s *t*-test (*: *p* < 0.1; **: *p* < 0.01; ***: *p* < 0.001).

##
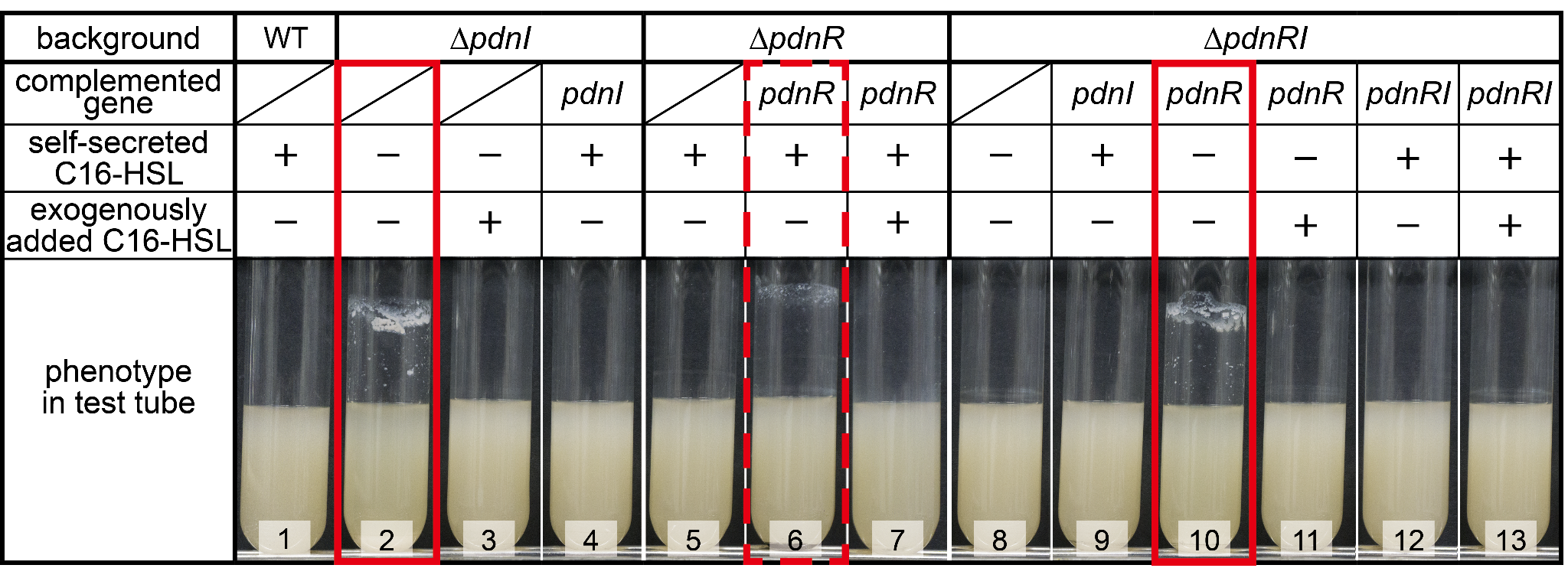
 Supplementary Figure S4. Images of test tube cultures of different strains with and without complementation of deleted gene(s). In the tubes where C16-HSL is present, it is either self-secreted when the strain possesses the *pdnI* gene or added exogenously. In the latter case, the concentration of C16-HSL is 5000 nM. The red boxes indicate the tubes with cell aggregation. The dashed red box indicates the specific case where the self-secreted quantity of C16-HSL is insufficient to fully suppress aggregation. All test tubes are incubated overnight with shaking at 30 ºC. These images are representative of biologically independent experiments (N=3).

**
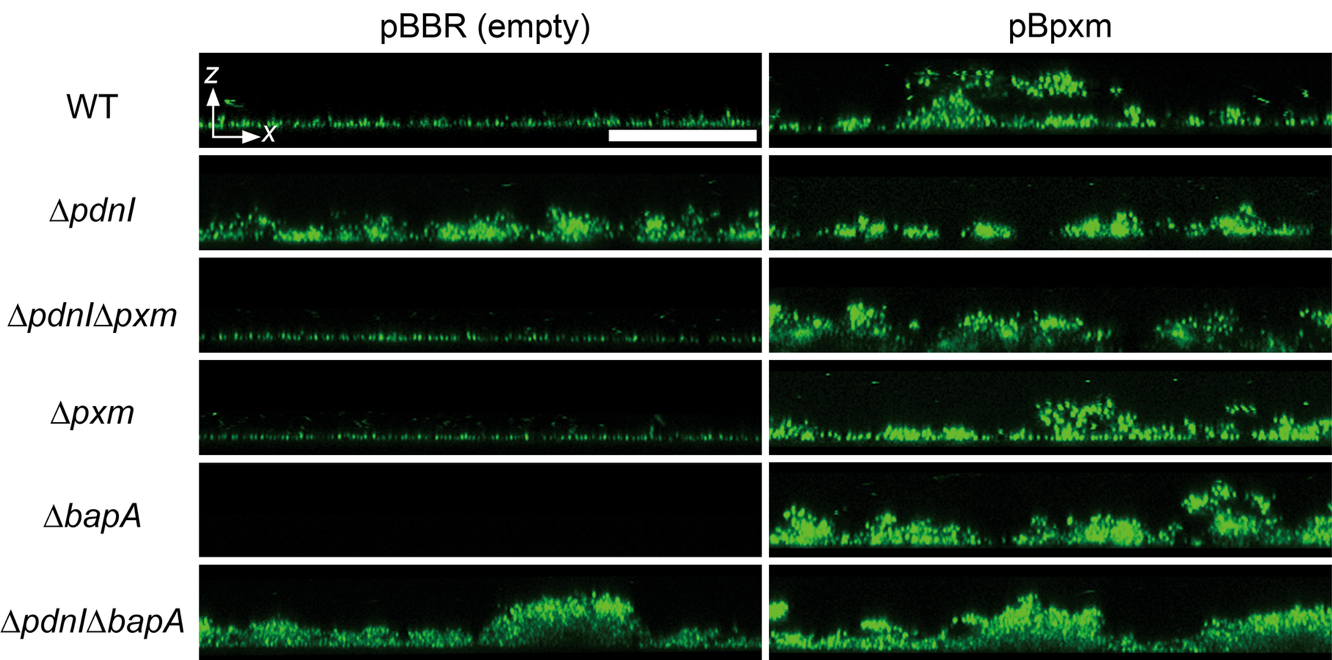
Supplementary Figure S5. Confocal images of WT and mutant strains carrying either pBBR or pBpxm.** The pBpxm plasmid drives constitutive overexpression of the *pxm* gene. Strains were cultured under static conditions for 48 h at 30 ºC, stained with SYTO9 (green), and imaged. Scale bar = 20 μm.


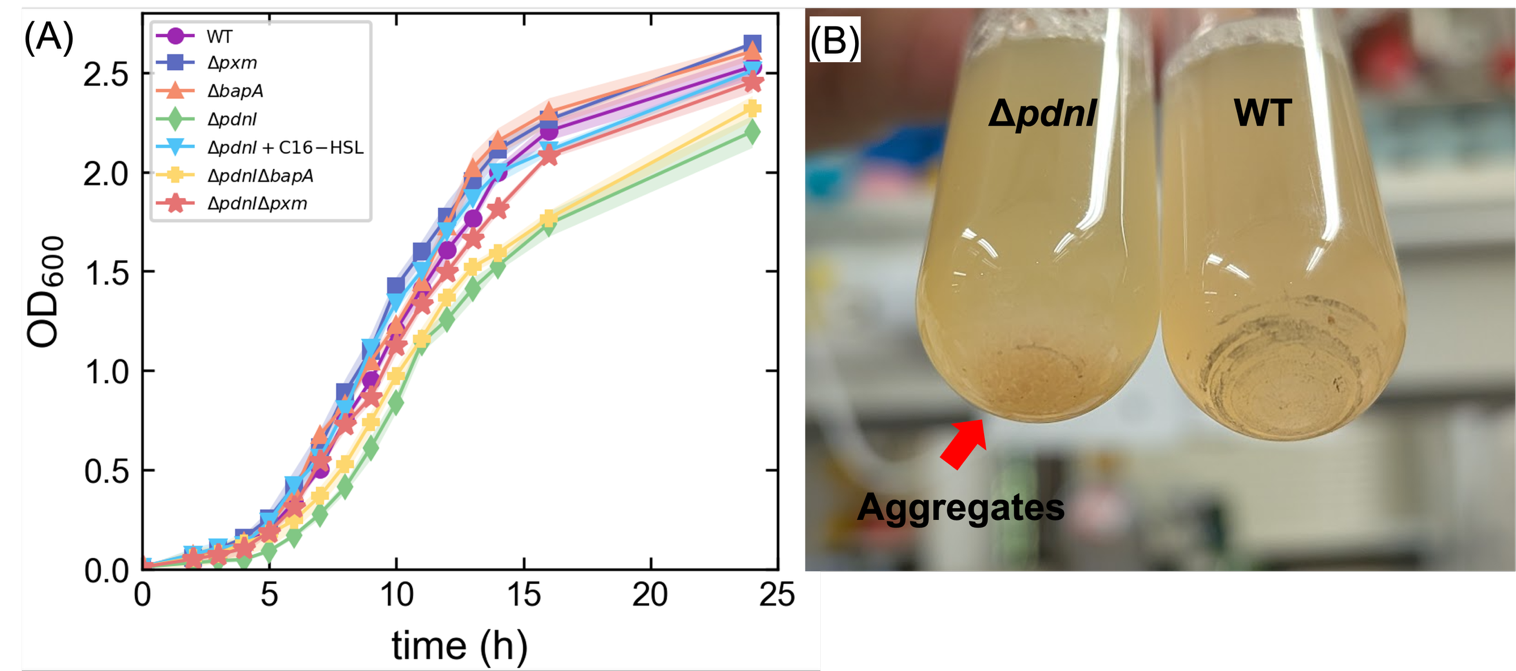


**Supplementary Figure S6.** (A) Growth curves of the major strains used in this study. The symbols and filled regions represent the mean ± s.d. (N=3). Cells were cultivated at 30 °C under continuous shaking (190 rpm). (B) Photograph of test tubes containing Δ*pdnI* and WT cultures with the arrow indicating aggregates. Of all strains, we observed aggregates in only Δ*pdnI* and Δ*pdnI*Δ*bapA* cultures.

**Supplementary Movie 1,** This movie shows *P. denitrificans* WT cells escaping from microcolonies under semi-confinement in the 2.0 μm tall chamber.

<https://www.dropbox.com/scl/fi/p9a6vfz285z561e8wrs8i/250504_MovieS1_v3.mov?rlkey=n9k42gnb7cifstoxwm6f0m44y&dl=0>

**Supplementary Movie 2,** This movie shows *P. denitrificans* WT colony expansion for all strains under confinement in the 1.3 μm tall chamber.

<https://www.dropbox.com/scl/fi/g5xtjkou6zurfizd1dvtf/250504_MovieS2_v3.mov?rlkey=h0j6i77kn8u9a8f5i6bdoy4pk&dl=0>

# Supplementary References

[1.    Toyofuku M, Morinaga K, Hashimoto Y, Uhl J, Shimamura H, Inaba H, et al. Membrane vesicle-mediated bacterial communication. *ISME J* 2017; **11**: 1504–1509.](https://sciwheel.com/work/bibliography/14669134)

[2.    Zhang Y, Gao J, Wang L, Liu S, Bai Z, Zhuang X, et al. Environmental Adaptability and Quorum Sensing: Iron Uptake Regulation during Biofilm Formation by *Paracoccus denitrificans*. *Appl Environ Microbiol* 2018; **84**.](https://sciwheel.com/work/bibliography/14672529)

[3.    Wang N, Gao J, Xiao S, Zhuang G. Overexpression of *pdeR* promotes biofilm formation of *Paracoccus denitrificans* by promoting ATP production and iron acquisition. *Front Microbiol* 2022; **13**: 966976.](https://sciwheel.com/work/bibliography/17840018)

[4.    Simon R, Priefer U, Pühler A. A broad host range mobilization system for *in vivo* genetic engineering: transposon mutagenesis in gram negative bacteria. *Nat Biotechnol* 1983; **1**: 784–791.](https://sciwheel.com/work/bibliography/2068593)

[5.    Shearer N, Hinsley AP, Van Spanning RJ, Spiro S. Anaerobic growth of *Paracoccus denitrificans* requires cobalamin: characterization of *cobK* and *cobJ* genes. *J Bacteriol* 1999; **181**: 6907–6913.](https://sciwheel.com/work/bibliography/14650228)

[6.    de Vries GE, Harms N, Hoogendijk J, Stouthamer AH. Isolation and characterization of *Paracoccus denitrificans* mutants with increased conjugation frequencies and pleiotropic loss of a (nGATCn) DNA-modifying property. *Arch Microbiol* 1989; **152**: 52–57.](https://sciwheel.com/work/bibliography/10111375)

[7.    O’Toole GA, Kolter R. Initiation of biofilm formation in *Pseudomonas fluorescens* WCS365 proceeds via multiple, convergent signalling pathways: a genetic analysis. *Mol Microbiol* 1998; **28**: 449–461.](https://sciwheel.com/work/bibliography/229693)

[8.    Schäfer A, Tauch A, Jäger W, Kalinowski J, Thierbach G, Pühler A. Small mobilizable multi-purpose cloning vectors derived from the *Escherichia coli* plasmids pK18 and pK19: selection of defined deletions in the chromosome of *Corynebacterium glutamicum*. *Gene* 1994; **145**: 69–73.](https://sciwheel.com/work/bibliography/4217473)

[9.    Simon R, O’Connell M, Labes M, Pühler A. Plasmid vectors for the genetic analysis and manipulation of rhizobia and other gram-negative bacteria. *Plant Molecular Biology*. 1986; pp 640–659.](https://sciwheel.com/work/bibliography/4941718)

[10.   Yoshida K, Toyofuku M, Obana N, Nomura N. Biofilm formation by *Paracoccus denitrificans* requires a type I secretion system-dependent adhesin BapA. *FEMS Microbiol Lett* 2017; **364**.](https://sciwheel.com/work/bibliography/14650241)

[11.   Kovach ME, Elzer PH, Hill DS, Robertson GT, Farris MA, Roop RM, et al. Four new derivatives of the broad-host-range cloning vector pBBR1MCS, carrying different antibiotic-resistance cassettes. *Gene* 1995; **166**: 175–176.](https://sciwheel.com/work/bibliography/2068591)
